# Reduction of Systematic Bias in Transcriptome Data from Human Peripheral Blood Mononuclear Cells for Transportation and Biobanking

**DOI:** 10.1371/journal.pone.0104283

**Published:** 2014-08-07

**Authors:** Hideki Ohmomo, Tsuyoshi Hachiya, Yu Shiwa, Ryohei Furukawa, Kanako Ono, Shigeki Ito, Yoji Ishida, Mamoru Satoh, Jiro Hitomi, Kenji Sobue, Atsushi Shimizu

**Affiliations:** 1 Division of Biobank and Data Management, Iwate Tohoku Medical Megabank Organization, Iwate Medical University, Shiwa-gun, Iwate, Japan; 2 Division of Biomedical Information Analysis, Iwate Tohoku Medical Megabank Organization, Iwate Medical University, Shiwa-gun, Iwate, Japan; 3 Division of Hematology and Oncology, Department of Internal Medicine, Iwate Medical University School of Medicine, Morioka, Iwate, Japan; 4 Division of Cardioangiology, Department of Internal Medicine and Memorial Heart Center, Iwate Medical University School of Medicine, Morioka, Iwate, Japan; 5 Department of Anatomy, Iwate Medical University School of Medicine, Shiwa-gun, Iwate, Japan; 6 Deputy Executive Director, Iwate Tohoku Medical Megabank Organization, Iwate Medical University, Shiwa-gun, Iwate, Japan; 7 Department of Neuroscience, Institute for Biomedical Sciences, Iwate Medical University, Shiwa-gun, Iwate, Japan; 8 Executive Director, Iwate Tohoku Medical Megabank Organization, Iwate Medical University, Shiwa-gun, Iwate, Japan; Ecole Normale Supérieure de Lyon, France

## Abstract

Transportation of samples is essential for large-scale biobank projects. However, RNA degradation during pre-analytical operations prior to transportation can cause systematic bias in transcriptome data, which may prevent subsequent biomarker identification. Therefore, to collect high-quality biobank samples for expression analysis, specimens must be transported under stable conditions. In this study, we examined the effectiveness of RNA-stabilizing reagents to prevent RNA degradation during pre-analytical operations with an emphasis on RNA from peripheral blood mononuclear cells (PBMCs) to establish a protocol for reducing systematic bias. To this end, we obtained PBMCs from 11 healthy volunteers and analyzed the purity, yield, and integrity of extracted RNA after performing pre-analytical operations for freezing PBMCs at −80°C. We randomly chose 7 samples from 11 samples individually, and systematic bias in expression levels was examined by real-time quantitative reverse transcription polymerase chain reaction (qRT-PCR), RNA sequencing (RNA-Seq) experiments and data analysis. Our data demonstrated that omission of stabilizing reagents significantly lowered RNA integrity, suggesting substantial degradation of RNA molecules due to pre-analytical freezing. qRT-PCR experiments for 19 selected transcripts revealed systematic bias in the expression levels of five transcripts. RNA-Seq for 25,223 transcripts also suggested that about 40% of transcripts were systematically biased. These results indicated that appropriate reduction in systematic bias is essential in protocols for collection of RNA from PBMCs for large-scale biobank projects. Among the seven commercially available stabilizing reagents examined in this study, qRT-PCR and RNA-Seq experiments consistently suggested that RNALock, RNA/DNA Stabilization Reagent for Blood and Bone Marrow, and 1-Thioglycerol/Homogenization solution could reduce systematic bias. On the basis of the results of this study, we established a protocol to reduce systematic bias in the expression levels of RNA transcripts isolated from PBMCs. We believe that these data provide a novel methodology for collection of high-quality RNA from PBMCs for biobank researchers.

## Introduction

On Friday, March 11, 2011, the northern part of Japan was affected by a magnitude-9.0 earthquake, termed the Great East Japan Earthquake. This event was the fourth most powerful earthquake on record and triggered a devastating tsunami. After the disaster, the incidence of cardiovascular and psychiatric diseases increased in the coastal areas of Iwate [1], [2] and Miyagi prefectures [3], [4], and a large-scale population-based biobank, the Tohoku Medical Megabank, was instituted for systematic data collection, storing, and parceling of biological samples, such as serum, plasma, peripheral blood mononuclear cells (PBMCs), and genomic DNA and RNA extracted from PBMCs and urine, as well as heath information (lifestyle, history of illness, and living environment) and gene analysis information, in order to investigate the effects of the earthquake and tsunami on the health of local residents.

Collection of blood RNA in population-based cohort studies provides an invaluable opportunity within research communities to search for blood RNA biomarkers for assessing risks associated with the onset of common diseases. Quality management of sample transportation from remote assessment centers to the central laboratory is essential for biobank projects. Although genomic DNA is stable against pre-analytical operations such as freezing and transportation [5], [6], RNA is less stable to conformational changes than DNA because RNA is subjected to spontaneous degradation at room temperature. To collect stabilized RNA from whole blood samples, the UK biobank (http://www.ukbiobank.ac.uk/) employs Tempus Blood RNA tubes (Life Technologies, Carlsbad, CA, USA) [7], while other biobank projects use the PAXgene RNA System (Becton Dickinson and Company, Franklin Lakes, NJ, USA) [8]. Whole blood components, such as plasma and heme-containing red blood cells, inhibit polymerase chain reaction (PCR) and reverse transcription [9], and high concentrations of globin mRNA in whole blood RNA interfere with accurate transcriptome analysis [10]. However, using PAXgene [11], [12] or Tempus [13] tubes can help to stabilize RNA profiles in whole blood. Some reports using these blood collection tubes have shown that globin reduction can alter the expression of several genes [13]–[15]. Therefore, PBMCs are commonly used for gene expression profiling, and clinical transcriptional studies can provide important findings for potential biomarkers of disease initiation [16], [17].

Here, we propose a protocol to minimize the effects of pre-analytical procedures on the transcriptome of PBMCs by assessing changes in the transcriptome with and without the use of stabilizing reagents. Seven commercially available stabilizing reagents were considered. Based on qRT-PCR experiments, we carried out quantitative analysis of gene expression for 19 selected candidate genes. Subsequently, we performed whole transcriptome analysis using a HiSeq2500 sequencer (Illumina Inc., San Diego, CA, USA) to examine changes in gene expression under various sample conditions. From our data, we developed a standard protocol for collecting high-quality RNA derived from PBMCs.

## Materials and Methods

### Ethics statement

Ethical approval for the study was obtained from the Ethical Committee of Iwate Medical University (Approval ID: HG H25-1). All the subjects gave us written informed consent to participate this study and the use of anonymous samples, explicitly explained to participants.

### Blood collection and pre-analytical operations

**Figure 1:**
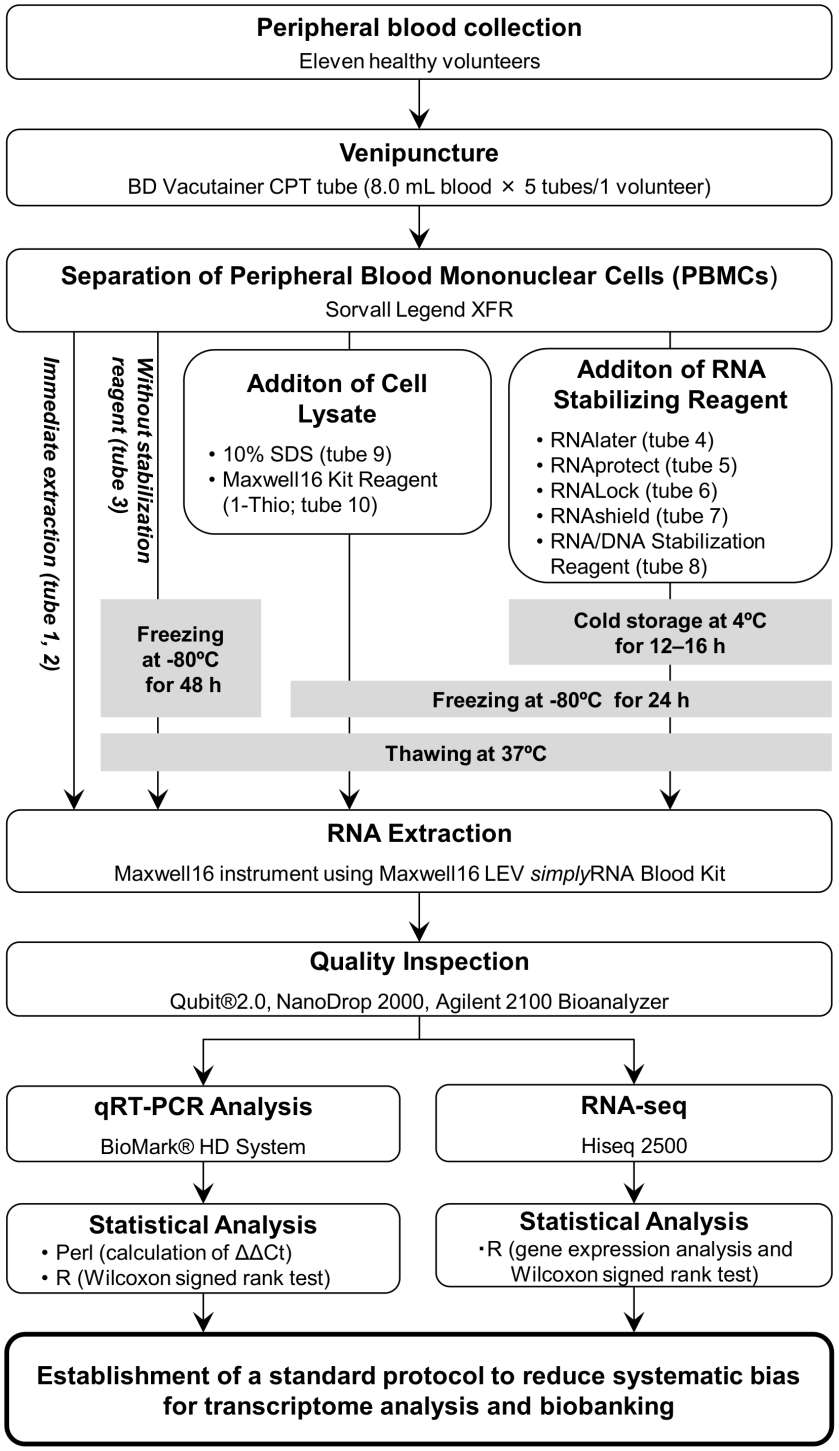
Workflow of the study design.

The workflow for this study is shown in Figure 1. Forty milliliters of whole blood was collected from 11 healthy volunteers (mean age, 35.3±10.6 years; eight men and three women) into five BD Vacutainer CPT tubes with sodium heparin (8 mL; Becton Dickinson and Company) through a 21-gauge needle. PBMCs were separated by centrifugation (Sorvall Legend XFR; Thermo Fisher Scientific, Waltham, MA) at 1,700×*g* for 20 min at room temperature. To remove any contaminating platelets and plasma, PBMCs were washed three times in 30 mL phosphate buffer saline (PBS) containing 2 mM EDTA, followed by centrifugation at 250×*g* for 10 min at room temperature [18]. PBMCs were then resuspended in 1 mL PBS, and cell numbers were determined using a C-Chip disposable hemocytometer (Biochrom AG, Berlin, Germany).

Collected PBMCs were dispensed into 10 tubes in 100 µL increments, and the mean PBMCs contained in each tube was 1.3×10^6^±5.0×10^5^ cells/mL. RNA from PBMCs in tubes 1 and 2 were extracted immediately (Ctrl1 and Ctrl2). PBMCs in tube 3 were immediately frozen at −80°C and stored for 48 h (termed the “Without stab” sample to indicate the lack of stabilizing reagent). The remaining aliquots of PBMCs were treated with the following reagents: tube 4, added to 1 mL RNAlater (Qiagen, Hilden, Germany; termed “Later”); tube 5, added to 500 µL RNAprotect (Qiagen; termed “Protect”); tube 6, added to 300 µL RNALock (Omega Bio-Tek, Doraville, GA, USA; termed “Lock”); tube 7, added to 400 µL RNAshield (Zymo Research, Orange, CA, USA; termed “Shield”); tube 8, added to 100 µL RNA/DNA Stabilization Reagent for Blood/Bone Marrow (Roche Diagnostics, Deutschland, GmbH; termed “Stab”); tube 9, added to 25 µL 10% sodium dodecyl sulfate (Sigma-Aldrich, St. Louis, MO, USA; termed “SDS”); and tube 10, added to 200 µL of a reagent provided in the Maxwell16 LEV *simply*RNA Blood Kit containing 1-Thioglycerol/Homogenization (Promega, Madison, WI, USA; termed “1-Thio”). Tubes 4–8 were frozen at −80°C over 24 h after storing at 4°C for 12–16 h. Tubes 9 and 10 were stored under conditions similar to those of tube 3.

### RNA extraction and measurement of purity, yield, and integrity

After thawing frozen PBMC samples at 37°C, total RNA was immediately extracted using a Maxwell16 LEV *simply*RNA Blood Kit on a Maxwell16 Instrument (Promega) according to the manufacturer’s instructions. A 4-µL aliquot of each sample of extracted total RNA was used for RNA quality control assessments, while the remaining RNA sample was stored at −80°C until use. The RNA yield was measured using a Qubit RNA Assay Kit on a Qubit2.0 Fluorometer (Life Technologies). RNA purity was estimated by measuring the ratio of absorbance at 260 and 280 nm (A260/A280) using a Nanodrop 2000 UV-Vis Spectrophotometer (Thermo Fisher Scientific). The RNA integrity number (RIN) was assessed using an RNA 6000 Nano Kit on an Agilent 2100 Bioanalyzer (Agilent Technologies, Santa Clara, CA, USA) according to the manufacturer’s instructions.

### Quantitative real time-PCR and data analysis

RNA expression levels were measured using TaqMan Gene Expression Assays (Applied Biosystems). We prepared TaqMan PCR primers and probes for 21 genes (Table 1). *GAPDH* and *ACTB* were selected as controls (also referred to as reference transcripts). Twelve genes (*IL1B*, *IL8*, *IL10*, *IL15*, *IFNG*, *TNF*, *JUN*, *HSPA1A*, *FOS*, *NFKB1*, *TP53*, and *NOS3*) were selected according to previous studies of RNA stability [19]. *SPI1* and *CSF1R* were selected as monocyte-specific markers. The remaining five genes were selected because these genes have been suggested to be biomarkers of human disease (*IL6*, *SLC6A4*, *HDAC5*, *NR3C1*, and *BDNF*).

**Table 1:** TaqMan probes for the 21 candidate genes analyzed by qRT-PCR.

Gene symbol	Major alias	Gene name	TaqMan probe ID
GAPDH	–	Glyceraldehyde-3-phosphate dehydrogenase	Hs99999905_m1
ACTB	β-Actin	Actin, beta	Hs99999903_m1
IL1B	IL-1β	Interleukin 1 beta	Hs00174097_m1
IL8	IL-8	Interleukin 8	Hs00174103_m1
IL10	IL-10	Interleukin 10	Hs00174086_m1
IL15	IL-15	Interleukin 15	Hs01003716_m1
IFNG	INF-γ	Interferon gamma	Hs00174143_m1
TNF	TNFα	Tumor necrosis factor	Hs00174128_m1
JUN	c-jun	Jun proto-oncogene	Hs99999141_s1
HSPA1A	HSP70	Heat shock 70-kDa protein 1A	Hs00359163_s1
FOS	c-fos	FBJ murine osteosarcoma viral oncogene homolog	Hs01119267_g1
NFKB1	NFκβ	Nuclear factor of kappa light polypeptide gene enhancer in B-cells 1	Hs00231653_m1
TP53	p53	Tumor protein p53	Hs00153340_m1
NOS3	ENOS	Nitric oxide synthase 3, endothelial cell	Hs01574659_m1
SPI1	PU.1	Spleen focus-forming virus (SFFV) proviral integration oncogene	Hs00231368_m1
CSF1R	CD115	Colony-stimulating factor 1 receptor	Hs00911250_m1
IL6	IL-6	Interleukin 6	Hs00174131_m1
SLC6A4	–	Serotonin transporter	Hs00984349_m1
HDAC5	–	Histone deacetylase 5	Hs00608366_m1
NR3C1	–	Glucocorticoid receptor	Hs00353740_m1
BDNF	–	Brain-derived neurotrophic factor	Hs00542425_s1

cDNA was synthesized from RNA samples using a SuperScript VILO cDNA Synthesis Kit (Life Technologies). For a combination of 48 samples×48 pairs of PCR primers, pre-amplification of cDNA and qRT-PCR experiments were performed in parallel BioMark 48.48 Dynamic Array chips (Fluidigm, South San Francisco, CA, USA). Two replicates of threshold cycle (Ct) values were measured for each condition.

The Ct values were analyzed using an in-house Perl script based on the ΔΔCt method [20]. Let *Ct_i,j,k_* be a Ct value for a condition *i*, a transcript *j*, and a replicate *k*. In our script, at first, *Ct_i,j_* = *Average_k_*(*Ct_i,j,k_*) was calculated by averaging Ct values over replicates. Next, Δ*Ct_i,j_* =  *Ct_i,j_*– *Ct_i,j_*_′_ was calculated by subtracting the Ct of the reference transcript *j*′ (e.g., *GAPDH* or *ACTB*) from the Ct of a target transcript *j*. Finally, ΔΔ*Ct_i,j_* = Δ*Ct_i,j–_*Δ*Ct_i_*_′,*j*_ was calculated by subtracting the ΔCt of the reference condition *i*′ (e.g., Ctrl1) from the ΔCt of the target condition *i*.

### Preparation of cDNA libraries and RNA-seq

We randomly chose seven of the 11 volunteers and the seven RNA samples were used for RNA-Seq. cDNA sequencing libraries were prepared from 250 ng of total RNA using a TruSeq RNA Sample Prep Kit v2 (Illumina) according to the manufacturer’s instructions. Briefly, a population of poly(A)+ RNA was selected and converted to a library of cDNA fragments (mean fragment length: 306–355 bp) with adaptors attached to both ends for sequencing. The fragment peak size of each library was assessed using D1K ScreenTape on an Agilent 2200 TapeStation (Agilent Technologies). The concentration of the libraries was quantified with qRT-PCR on a StepOnePlus (Life Technologies) using a Kapa Library Quantification Kit for the Illumina sequencing platform (Kapa Biosystems, Woburn, MA, USA). Based on the individual library concentrations, equimolar pools of four libraries were prepared at a concentration of 11 pM and verified by additional qRT-PCR analysis. The pooled libraries were then loaded into two flow cells for cluster generation using a TruSeq PE Cluster Kit v3-cBot-HS (Illumina) and sequenced on a HiSeq2500 system (Illumina) using a TruSeq SBS Kit v3-HS (200 cycles; Illumina), generating 2×101 bp reads.

### Data analysis of RNA-seq

Base calling and quality filtering were performed using real-time analysis (RTA) with HCS v2.0. The quality of sequence data was evaluated by FastQC (http://www.bioinformatics.babraham.ac.uk/projects/fastqc/). The human reference sequence file (hs37d5.fa) was downloaded from the 1000genome ftp site (ftp://ftp.1000genomes.ebi.ac.uk/vol1/ftp/technical/reference/phase2_reference_assembly_sequence/), and the annotated general feature format (gff) file was downloaded from the Illumina iGenome ftp site (ftp://igenome:G3nom3s4u@ussd-ftp.illumina.com/Homo_sapiens/NCBI/build37.2/). The human genome index was constructed with the bowtie-build v.0.12.9 [21]. The fastq files were aligned to the reference genomic sequence by TopHat v.2.0.9 [22] with samtools v.0.1.19 [23]. Estimation of transcript abundance was calculated, and the count values were normalized to the upper quartile of the fragments per kilobase of transcript per million fragments mapped reads (FPKM) using Cuffdiff v2.1.1 [24]. Since RNA from three individuals was analyzed by both qRT-PCR and RNA-Seq, the Spearman correlation coefficient between the results of qRT-PCR (ΔCt value) and RNA-Seq (log_10_[FPKM+1] value) was calculated by comparing 456 (3 individuals×8 conditions×19 transcripts) dimensional vectors. Pearson correlation coefficients between pre-analytical conditions were calculated based on log_10_[FPKM+1] values. Therefore, we first calculated the Pearson correlation coefficients between condition pairs for each of the seven individuals, and then the average and standard deviation of the correlation coefficients across the seven individuals were calculated for each condition pair. Cluster analysis was also performed based on log_10_[FPKM+1] values. A dendrogram was created using the CummeRbund package in R [25]. Transcripts that were differentially expressed among pre-analytical conditions were tested based on the Wilcoxon signed rank test.

## Results

### Effects of pre-analytical conditions on RNA yield, integrity, and purity

PBMCs from 11 volunteers were subjected to 10 pre-analytical conditions: Ctrl1, Ctrl2, Without stab, Later, Protect, Lock, Shield, Stab, SDS, and 1-Thio. The purity, yield, and integrity of extracted RNA for each of the 10 conditions are shown in Table 2.

**Table 2:** RNA purity, yield, and integrity.

Sample conditions	A260/A280	RNA yield (µg)	RIN
**RNA extraction immediately after venipuncture**			
Ctrl 1	1.96±0.06	2.91±1.44	9.56±0.22
Ctrl 2	1.96±0.07	3.00±1.51	9.59±0.25
**RNA extraction after freezing for over 24 h**			
Without stab	1.97±0.06	2.93±1.16	8.44±1.00**
Later	1.60±0.14***	0.31±0.08$ ^,^**	N.A.
Protect	1.92±0.05**	2.06±0.63	7.66±0.33***
Lock	1.99±0.04*	1.73±0.62*	9.57±0.31
Shield	1.87±0.10***	0.93±0.53# ^,^**	N.A.
Stab	2.02±0.04***	2.91±1.29	9.45±0.65
SDS	1.97±0.05	2.14±0.97*	5.93±1.93***
1-Thio	1.99±0.04**	2.56±0.98	9.74±0.17*

RNA purity, yield, and integrity were measured for 11 individual samples. A260/A280 indicates the ratio of absorbance at 260 and 280 nm.

N.A.: not available (not measureable), RIN: RNA integrity number.

*p<0.05; **p<0.01; ***p<0.001 (Wilcoxon signed rank test).

$ RNA yield could be measured for nine individual samples (failed in two samples).

# RNA yield could be measured for 10 individual samples (failed in one sample).

The RNA yield of the Ctrl2 condition was equivalent to that of the Ctrl1 condition, confirming that our experimental results were consistent between replicates. Moreover, the RNA yield of the Without stab condition was equivalent to that of the Ctrl1 condition. This result implied that pre-analytical freezing did not lower RNA yield. Similarly, the RNA yield of the Protect, Stab, and 1-Thio conditions were not significantly different from that of the Ctrl1 condition. In contrast, the RNA yield of the Lock, SDS, Later, and Shield conditions was significantly lower than that of the Ctrl1 condition (Table 2). These data indicated that there was insufficient RNA yield for biobanking purposes under these conditions.

The RNA integrity (RIN value) of the Ctrl2 condition was equivalent to that of the Ctrl1 condition. The RNA integrity of the Without stab condition was significantly lower than that of the Ctrl1 condition (Table 2). This result suggested that pre-analytical freezing significantly lowered RNA integrity, implying that addition of stabilizing reagents was essential for prevention of reduced RNA integrity. Under the Lock and Stab conditions, RNA integrity was equivalent to that of the Ctrl1 condition. The RNA integrity of the 1-Thio sample was higher than that of the Ctrl1 condition, while the RNA integrity under the Protect and SDS conditions was significantly lower than that of the Ctrl1 condition. For the Later and Shield conditions, RNA integrity could not be measured, possibly due to low RNA yield and concentration. These results implied that RNA degradation during pre-analytical operations was effectively prevented under the Lock, Stab, and 1-Thio conditions.

The RNA purity (i.e., A260/A280) under the Ctrl2 condition was equivalent to that of the Ctrl1 condition. Similarly, under the Without stab and SDS conditions, the difference in RNA purity compared to the Ctrl1 condition was not significant. The RNA purities under the Later, Protect, and Shield conditions were significantly lower than that of the Ctrl1 condition. Conversely, the RNA purities of the Lock, Stab, and 1-Thio conditions were improved compared to that of the Ctrl1 condition. These results indicated that stabilizing reagents could significantly affect the purity of RNA.

From these results, we excluded the Later and Shield conditions from candidate reagents for preventing RNA degradation because of their insufficient RNA yield. The other six conditions (Without stab, Protect, Lock, Stab, SDS, and 1-Thio) as well as the two control conditions (Ctrl1 and Ctrl2) were examined in subsequent quantitative expression analyses.

### Gene expression bias during pre-analytical operations analyzed by qRT-PCR

**Figure 2:**
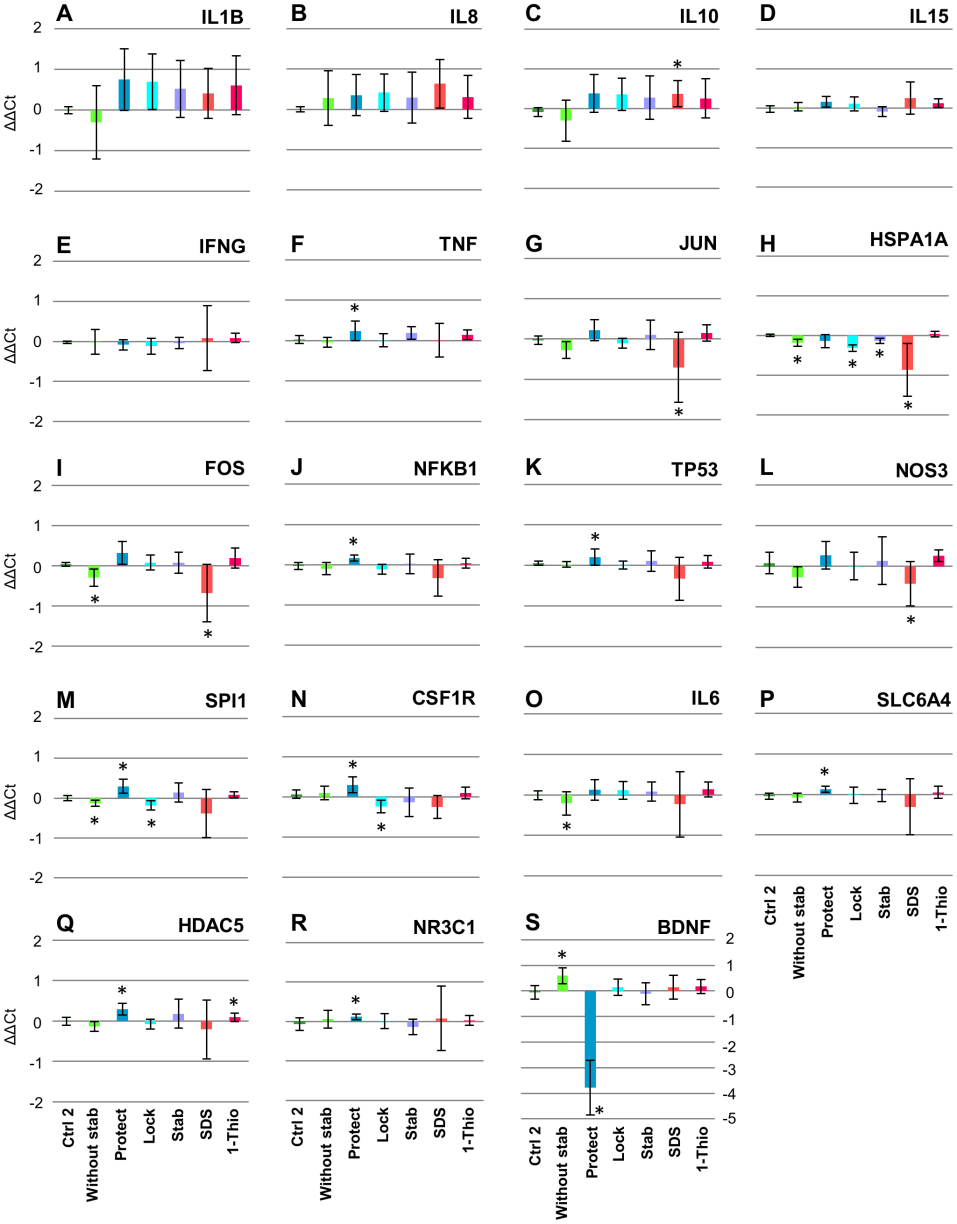
qRT-PCR analysis of 19 target transcripts. *GAPDH* and the Ctrl1 condition were used as a reference transcript and condition, respectively. ΔΔCt values are shown by vertical axes. Horizontal axes represent pre-analytical conditions in the following order: Ctrl2, Without stab, Protect, Lock, Stab, SDS, and 1-Thio. *, *p*<0.05; **, *p*<0.01 (Wilcoxon signed rank test).

To examine whether gene expression in PBMCs was biased during pre-analytical operations and whether the bias could be reduced using stabilizing reagents, we performed qRT-PCR assays to analyze the expression of 21 transcripts from seven individuals for each of the eight conditions. When *GAPDH* was used as control, no significant bias in ΔCt was observed between the Ctrl1 and Ctrl2 conditions for all target transcripts (Figure 2), suggesting that our experimental results were consistent between replicates. Between the Without stab and Ctrl1 conditions, five transcripts (*FOS*, *SPI1*, *IL6*, *HSPA1A*, and *BDNF*) were significantly biased (significance level of 0.05), indicating that absence of a stabilizing reagent promoted bias of transcript expression in a significant portion (∼40%) of transcripts. Under the Protect, Lock, Stab, SDS, and 1-Thio conditions, nine, three, one, five, and one transcripts were significantly biased, respectively, compared to the Ctrl1 condition (Figure 2). These results indicated that gene expression bias during pre-analytical operations was reduced under the Lock, Stab, and 1-Thio conditions. Similar results were obtained when *ACTB* was used as control (Figure S1).

### Pre-analytical operation bias in whole transcriptome analyzed by RNA-Seq

**Figure 3:**
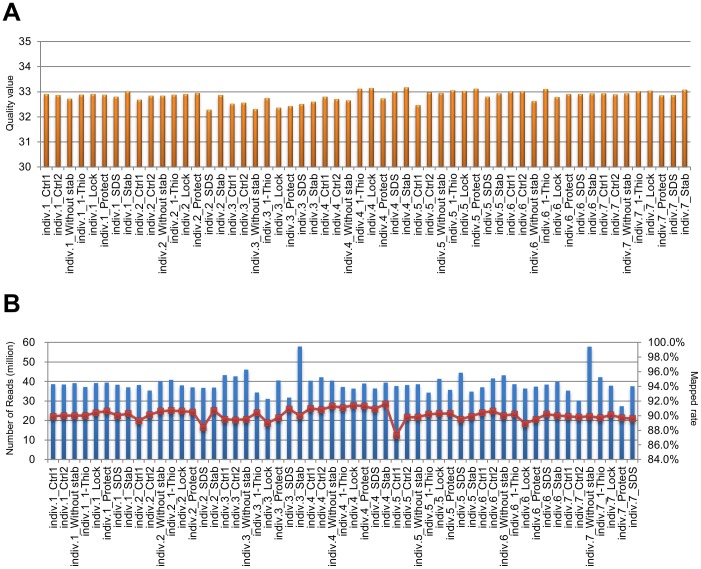
Quality assessment and control of sequencing data from HiSeq2500. **A.** Quality values of sequence reads calculated by Cufflinks (Cuffdiff) in each sample. **B.** The blue bars show the number of sequence reads mapped to the human genome (hs37d5) with TopHat, and the red line with squares indicates the mapped percentage in each sample (B).

To extend the above expression analysis of the 19 target transcripts to the whole transcriptome, we performed RNA-Seq for 56 samples (7 individuals×8 conditions; Figure 1). The base quality of 95–99 nucleotides was 32.8±0.2 (Figure 3A). The number of sequenced reads was 38.8±5.0 million. The percentage of mapped reads was 90.1% ±0.0% (Figure 3B). These statistics suggested that our sequencing data had sufficient quality for expression analyses.

**Figure 4:**
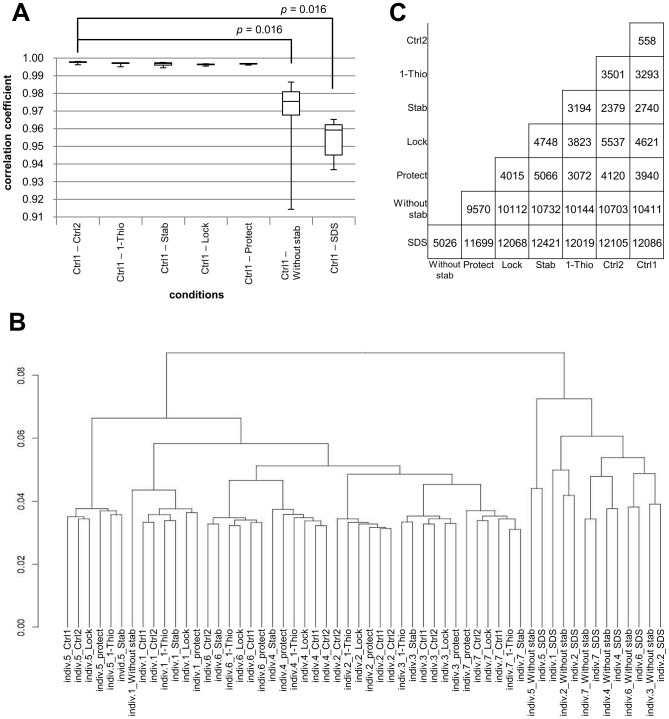
Data bias of transcriptome analysis in each condition. **A.** Correlation analysis of the average of FPKM under eight conditions for each sample. **B.** Cluster analysis of 56 transcriptomes: eight conditions for each of seven volunteers. **C.** Pair-wise comparisons of significant differences in gene expression for each sample. The number in each box shows the number of differentially expressed genes (*p*<0.05, Wilcoxon signed rank test).

As an indicator of normalized gene expression levels, FPKM values were calculated using TopHat and Cufflinks (File S1) [22], [23], [24]. Spearman correlation coefficient between Δ*Ct* values measured by qRT-PCR experiments (*GAPDH* was used as a reference transcript) and the FPKM value calculated from RNA-Seq data was −0.762 (*P*<2.2×10^−16^), suggesting that qRT-PCR and RNA-Seq showed consistent quantitative results (Figure S2). Figure 4A shows the average and standard deviation of Pearson correlation coefficients between the Ctrl1 conditions and the seven other conditions. Importantly, the Pearson correlation coefficient between the Ctrl1 and Ctrl2 conditions was 0.9955±0.00057, indicating the markedly high reproducibility of our experimental results between replicates. Additionally, the Pearson correlation coefficient between the Without stab and Ctrl1 conditions was 0.9825±0.010, which was significantly lower than that between the Ctrl1 and Ctrl2 conditions (*P* = 0.016; Wilcoxon signed rank test). This result suggested that pre-analytical freezing induced a systematic bias in the expression level of the whole transcriptome. As expected, under the 1-Thio, Stab, Lock, and Protect conditions, Pearson correlation coefficients with the Ctrl1 condition were not significantly different from that between the Ctrl1 and Ctrl2 conditions (Figure 4A). However, the Pearson correlation coefficient between the SDS and Ctrl1 conditions was significantly lower than that between the Ctrl1 and Ctrl2 conditions (*P* = 0.016; Wilcoxon signed rank test). These results showed that the 1-Thio, Stab, Lock, and Protect conditions could reduce systematic bias due to pre-analytical operations. Pair-wise scatter plots between the eight conditions are shown in .

To examine whether the transcriptomic bias due to pre-analytical operations was larger than transcriptomic variations between individuals, we performed clustering analysis of the 56 transcriptomes (Figure 4B). Interestingly, the Ctrl1, Ctrl2, 1-Thio, Stab, Lock, and Protect conditions were clustered together for each individual. The Without stab and SDS conditions were not included in the individual clusters. These results indicated that transcriptomic bias under the 1-Thio, Stab, Lock, and Protect conditions was less drastic than transcriptomic variations between individuals, whereas transcriptomic bias under the Without stab and SDS conditions was larger than transcriptomic variations between individuals.

We detected differentially expressed transcripts (DETs) between conditions by testing whether FPKM values under a condition were different from those under another condition using the Wilcoxon signed rank test with a significance level of 0.05 (Figure 4C). Of 25,223 transcripts, only 558 transcripts were detected as DETs between the Ctrl1 and Ctrl2 conditions. The number of DETs was within the expected number of false-positive discoveries (25,223×0.05 = 1,261). The number of DETs between the Without stab and Ctrl1 conditions was 10,411, which was much larger than the expected number of false-positive discoveries. Under the 1-Thio, Stab, Lock, and Protect conditions, the numbers of DETs were 3,293, 2,740, 4,621, and 3,940, respectively. These results suggested that the stabilizing reagents tested in this study could decrease systematic bias in FPKM values, although the number of DETs was larger than the expected number of false-positive discoveries. The number of DETs between the SDS and Ctrl1 conditions was 12,086, indicating that SDS increased rather than reduced systematic bias in FPKM values. To take into account not only the number of DETs, but also the *P*-value distribution, we drew quantile-quantile plots (QQ-plots, Figure S4). The *P*-value distributions of the 1-Thio, Stab, Lock, and Protect conditions were more similar to that of the Ctrl2 condition compared to those of the Without stab and SDS conditions.

## Discussion

In this study, we examined the effects of pre-analytical freezing, which is essential for the collection of PBMCs from remote assessment centers to a central laboratory, on gene expression levels in a transcriptome-wide manner. Even when no stabilizing reagent was used (i.e., the “Without stab” condition), RNA yield and purity were not lowered by pre-analytical freezing. However, RNA integrity was lowered under the Without stab condition, indicating that substantial degradation of RNA molecules occurred. qRT-PCR analysis supported this conclusion, demonstrating that five out of 19 transcripts exhibited systematic bias under the Without stab condition. In addition, whole-transcriptome analysis suggested that the expression levels of 10,411 out of 25,223 transcripts were systematically biased, suggesting that approximately 40% of whole transcripts were systematically biased due to pre-analytical freezing. Accordingly, it is necessary to implement an appropriate protocol to reduce systematic bias in gene expression levels to collect RNA from PBMCs for large-scale biobanks.

In this study, we analyzed the effects of seven commercially available stabilization reagents (Later, Protect, Lock, Shield, Stab, SDS, and 1-Thio; see the Methods section) on RNA purity, integrity, and yield. Our results demonstrated that Later and Shield significantly lowered RNA yield, making these two conditions inappropriate for biobanking purposes. Of the five remaining candidate reagents, the reduced RNA integrity observed under the Without stab condition was improved under the Lock, Stab, and 1-Thio conditions. Furthermore, qRT-PCR and RNA-Seq experiments consistently confirmed that systematic bias in expression levels was substantially reduced under these three conditions. Thus, use of these reagents could substantially improve collection and analysis of RNA for biobanks.

**Figure 5:**
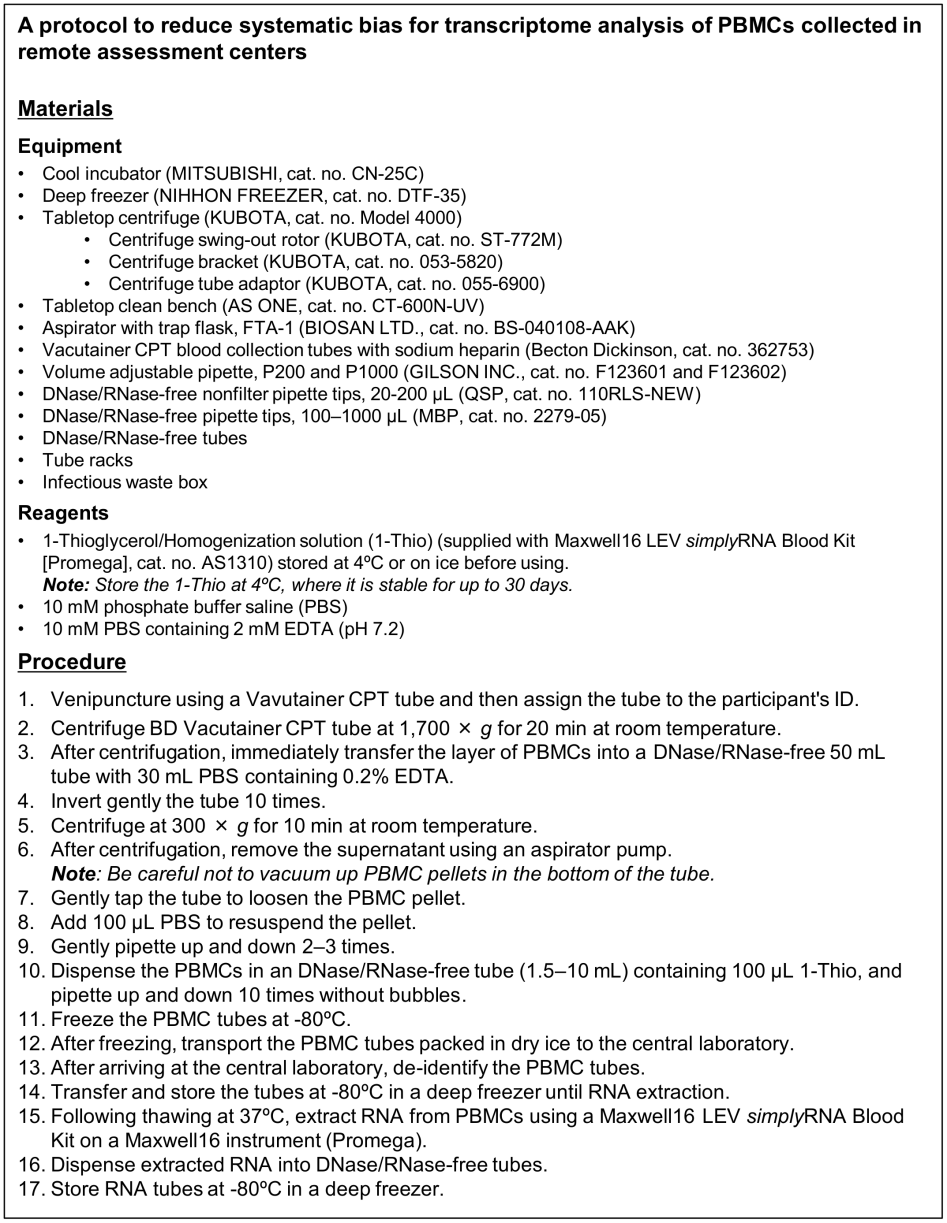
Protocol to reduce systematic bias for transcriptome analysis of PBMCs collected in remote assessment centers. A protocol for pre-analytical operations to mediate the effects of systematic bias in transcriptome data of PBMCs for transportation and biobanking is shown.

Protocols for sample collection for large-scale biobanks must be as simple and as low cost as possible. Based on the results of the present study, we established a protocol for pre-analytical treatment of samples at remote assessment centers to obtain high-quality RNA from PBMCs at a central laboratory (Figure 5). For blood collection (Procedure 1), we used a Vacutainer CPT tube, which could help to reduce contamination of red blood cells in the PBMC layer without the need for technical expertise. After centrifugation (Procedure 2), extracted PBMCs were washed with more than 15 volumes of 10 mM PBS containing 2 mM EDTA (pH 7.2) to remove platelets (Procedures 3–6). Then, PBMCs are resuspended in a suitable volume of 10 mM PBS (Procedures 7–9). We assumed that RNA extraction was performed using a Maxwell16 instrument at a central laboratory (Procedure 15). Considering the cost of reagents, we selected 1-Thio, which is supplied with the Maxwell16 LEV *simply*RNA Blood Kit, as a stabilizing reagent (Procedure 10). After this pretreatment, the tubes were frozen at −80°C for at least 24 h and at most one week (Procedure 11). For these pre-analytical operations, a cool incubator, a deep freezer, a tabletop centrifuge, a tabletop clean bench, and an aspirator are required at remote assessment centers. This equipment can be arranged within an area of 2×2 m.

Our protocol would require remote assessment centers to ship frozen tubes to a central laboratory using dry ice (Procedure 12). When the tubes would arrive at the central laboratory, they would be de-identified (Procedure 13) and would then be stored at −80°C until RNA extraction (Procedure 14). For RNA extraction, 32 tubes could be processed simultaneously. After thawing tubes at 37°C, RNA would be extracted from PBMCs using a Maxwell16 instrument (Procedure 15). The RNA liquid would be quantified and transferred into 4 sterile, barcoded cryovials (Procedure 16), which would then be stored separately in four deep freezers (Procedure 17). These procedures and barcoded samples could be managed using a robust and flexible LIMS system.

Our protocol would require two technical staff at each remote assessment center and two technical staff at the central laboratory. We trained all technical staff at the central laboratory using standardized training curriculum and a certification examination. Compared with the collection methods using PAXgene [11], [12] and Tempus [13] tubes, our protocol was more labor-intensive, and the cost of consumed goods was more expensive. Accordingly, there was a tradeoff between cost and quality in our protocol, and this tradeoff should be discussed for each biobank project. Our protocol presented in this study provided a novel methodology to collect high-quality RNA from PBMCs for biobank researchers. Collecting RNA from PBMCs based on our protocol may enable, for example, multilayer omics analysis (genome, methylome, and transcriptome) of PBMCs. We plan to collect 5,400 RNA samples according to our protocol over the next two years (2015–2016). In our feasibility trial, 32 RNA samples have been collected. Based on the trial, we modified and validated our protocol to make it as effective, manageable, and feasible as possible.

## Supporting Information

Figure S1 **Comparisons of alternative gene expression using the ΔΔCt method.** The reference gene was *ACTB*, and the reference sample was Ctrl1. The bar plot shows the mean ΔΔCt ± SD for each sample condition. *, *p*<0.05; **, *p*<0.01; ***, *p*<0.001 (Wilcoxon signed rank test).
(TIFF)

Figure S2 **Spearman correlation coefficient between ΔCt and log_10_[FPKM+1] in 19 genes.** ΔCt values measured by qRT-PCR experiments (using *GAPDH* as a reference transcript) are shown on the vertical axis. Log_10_[FPKM+1] values (calculated from RNA-Seq data) are shown on the horizontal axis. Each symbol has 24 data points representing 3 individuals×8 conditions. Spearman’s analysis between ΔCt and log_10_[FPKM+1] values showed consistent quantitative results.
(TIFF)

Figure S3 **Correlation analysis between results from eight conditions for seven volunteers.** Scatter plots on the bottom left compare the log_10_[FPKM+1] between the eight sample conditions. The histograms in the boxes show the number of expressed genes (diagonally from top left to bottom right). The numbers on the top right indicate the correlation coefficient value between each sample condition.
(TIFF)

Figure S4 **Quantile-quantile plots (QQ-plots) comparing the distribution of** ***P*** **-values from RNA-Seq.** The *P*-value was calculated by Wilcoxon signed rank test. The horizontal axis corresponds to the −log_10_(*P*-value) between the Ctrl2 and Ctrl1 conditions. The vertical axis corresponds to the −log_10_(*P*-value) between Stab (A), 1-Thio (B), Lock (C), Protect (D), SDS (E), Without stab. (F) and Ctrl1 conditions.
(TIFF)

File S1 **FPKM values for 25,223 genes from eight conditions for seven volunteers.**
(ZIP)
