# Supplementary figures and images for: Reduction of Systematic Bias in Transcriptome Data from Human Peripheral Blood Mononuclear Cells for Transportation and Biobanking
Source: PLoS One. 2014 Aug 7;9(8):e104283. doi: 10.1371/journal.pone.0104283 (PMC4125218; doi:10.1371/journal.pone.0104283)

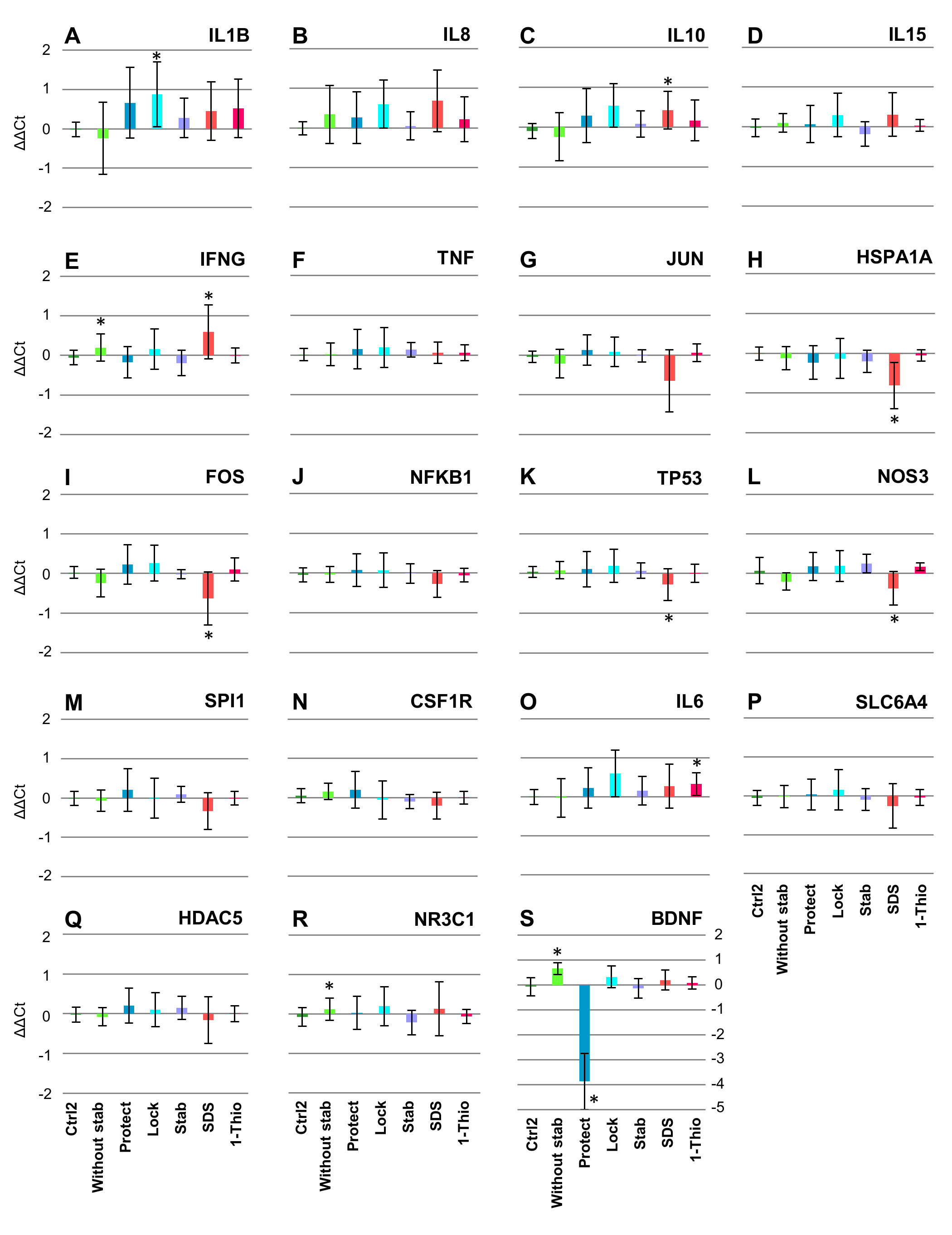

Supplement: Figure S1 — Comparisons of alternative gene expression using the ΔΔCt method. The reference gene was ACTB, and the reference sample was Ctrl1. The bar plot shows the mean ΔΔCt ± SD for each sample condition. *, p<0.05; **, p<0.01; ***, p<0.001 (Wilcoxon signed rank test). (TIFF) [file pone.0104283.s001.tiff]

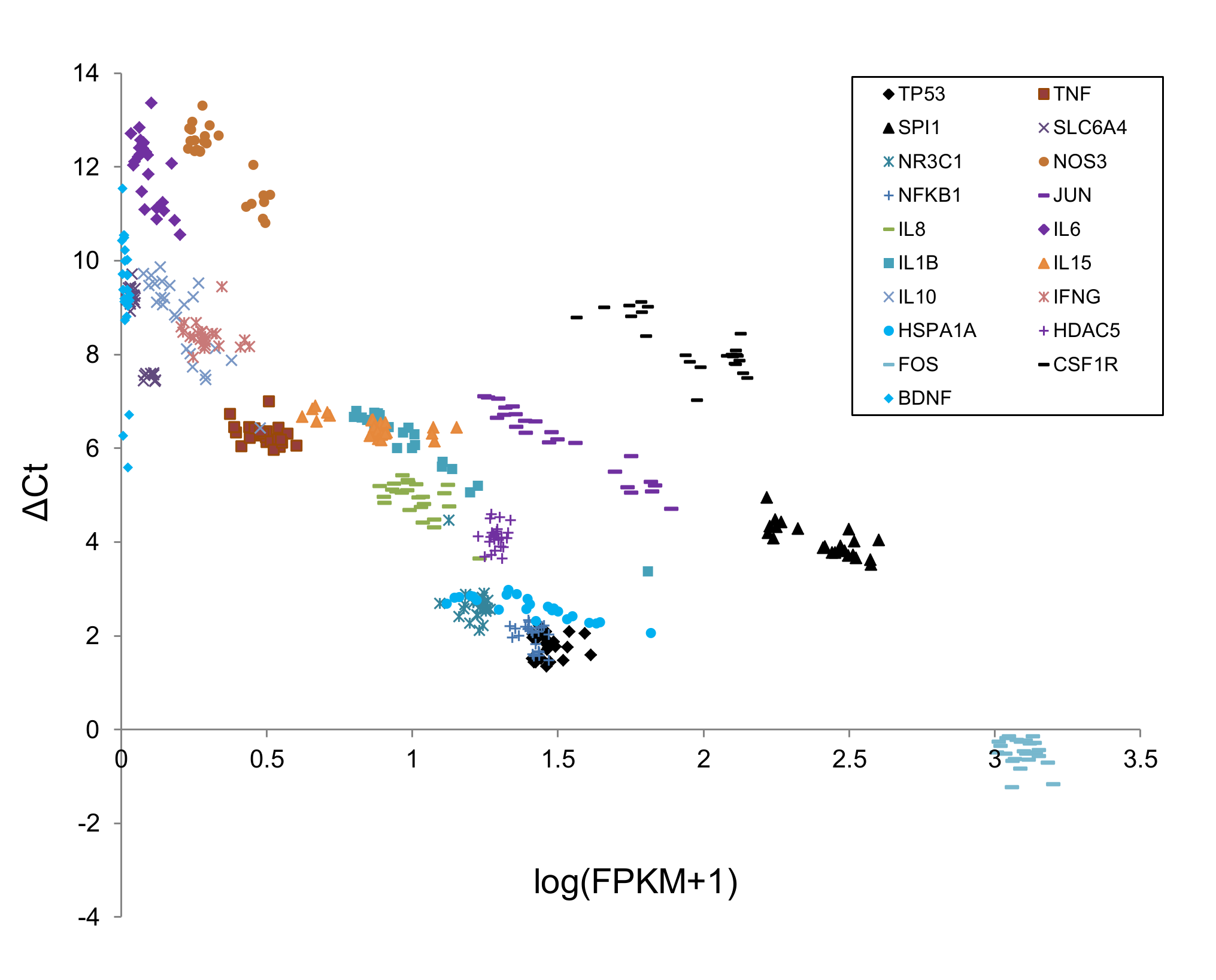

Supplement: Figure S2 — Spearman correlation coefficient between ΔCt and log10[FPKM+1] in 19 genes. ΔCt values measured by qRT-PCR experiments (using GAPDH as a reference transcript) are shown on the vertical axis. Log10[FPKM+1] values (calculated from RNA-Seq data) are shown on the horizontal axis. Each symbol has 24 data points representing 3 individuals×8 conditions. Spearman’s analysis between ΔCt and log10[FPKM+1] values showed consistent quantitative results. (TIFF) [file pone.0104283.s002.tiff]

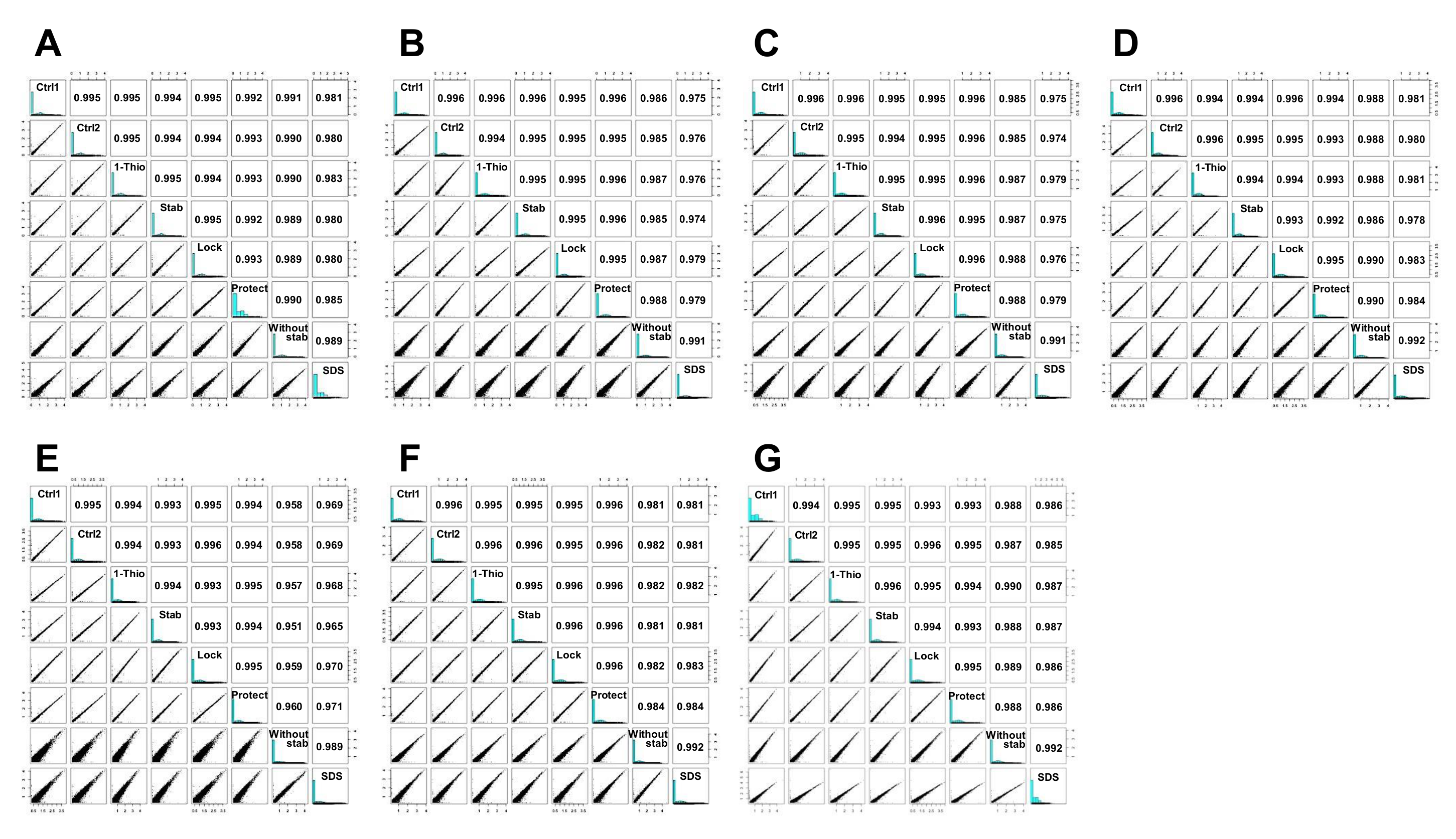

Supplement: Figure S3 — Correlation analysis between results from eight conditions for seven volunteers. Scatter plots on the bottom left compare the log10[FPKM+1] between the eight sample conditions. The histograms in the boxes show the number of expressed genes (diagonally from top left to bottom right). The numbers on the top right indicate the correlation coefficient value between each sample condition. (TIFF) [file pone.0104283.s003.tiff]

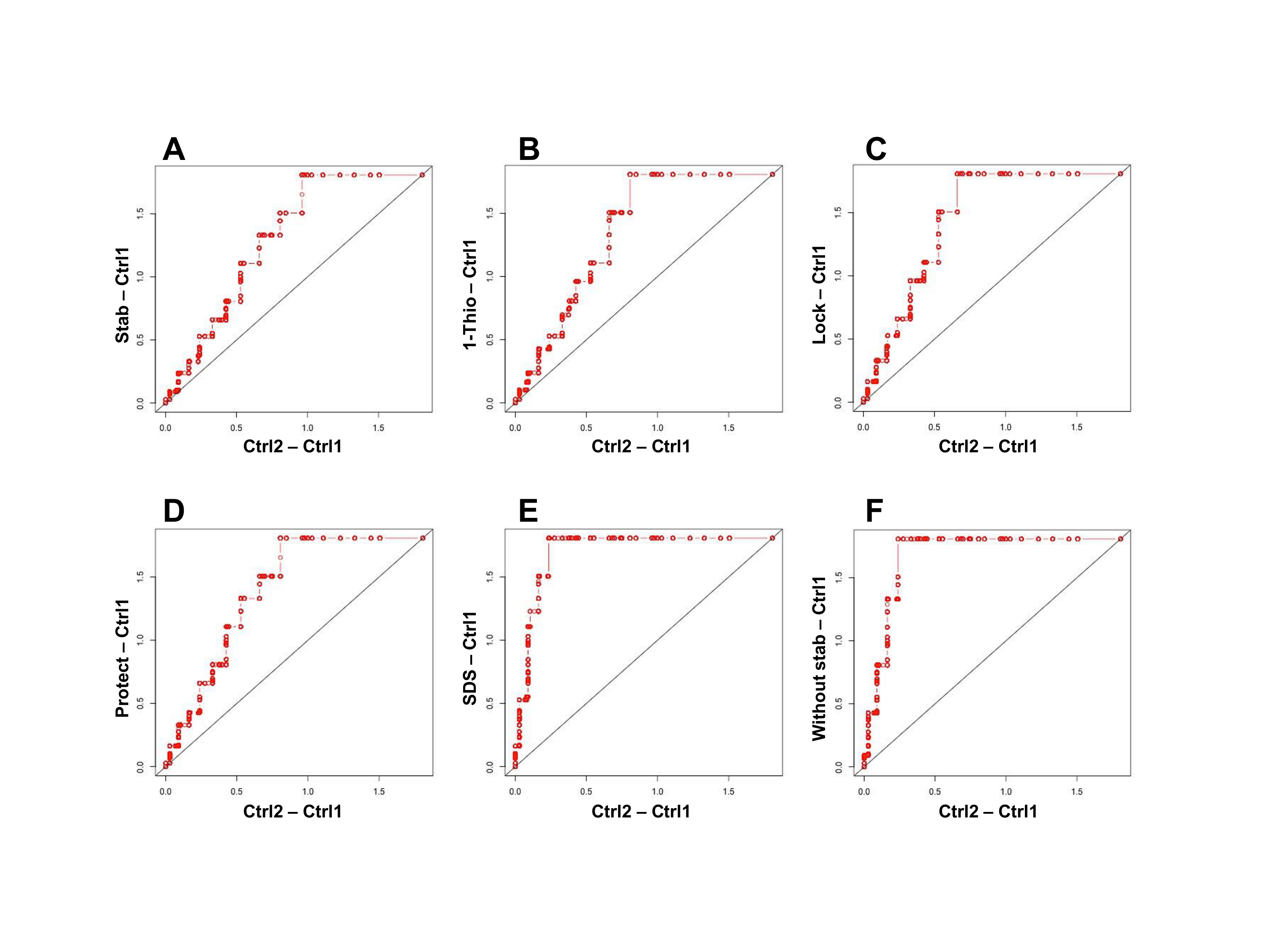

Supplement: Figure S4 — Quantile-quantile plots (QQ-plots) comparing the distribution of P -values from RNA-Seq. The P-value was calculated by Wilcoxon signed rank test. The horizontal axis corresponds to the −log10(P-value) between the Ctrl2 and Ctrl1 conditions. The vertical axis corresponds to the −log10(P-value) between Stab (A), 1-Thio (B), Lock (C), Protect (D), SDS (E), Without stab. (F) and Ctrl1 conditions. (TIFF) [file pone.0104283.s004.tiff]
